# Authors’ Reply: Reassessing AI in Medicine: Exploring the Capabilities of AI in Academic Abstract Synthesis

**DOI:** 10.2196/65123

**Published:** 2024-12-23

**Authors:** Tien-Wei Hsu, Chih-Sung Liang

**Affiliations:** 1 Department of Psychiatry E-Da Dachang Hospital I-Shou University Kaohsiung Taiwan; 2 Department of Psychiatry E-Da Hospital I-Shou University Kaohsiung Taiwan; 3 Department of Psychiatry Tri-service Hospital, Beitou Branch Taipei Taiwan; 4 Department of Psychiatry National Defense Medical Center Taipei Taiwan

**Keywords:** ChatGPT, AI-generated scientific content, plagiarism, AI, artificial intelligence, NLP, natural language processing, LLM, language model, text, textual, generation, generative, extract, extraction, scientific research, academic research, publication, abstract, comparative analysis, reviewer bias

We appreciate Wang and Zhou’s [1] keen interest in our publication [2]. Below are our responses to their comments.

First, Wang and Zhou [1] argued potential biases could arise from the 8 experts’ personal preferences, academic or cultural backgrounds, or their familiarity with the subject matter. However, the 30 papers we selected were from 3 different journals and explored various topics (as outlined in Table S3 in Multimedia Appendix 1 of our paper [2]). Therefore, these biases are unlikely to occur, considering the significant variation among the articles.

Second, we did not use ChatGPT 3.5. We used ChatPDF, which is based on ChatGPT 3.5 but can efficiently analyze PDF content.

Third, Wang and Zhou argued that if artificial intelligence (AI) assistance in refining an article does not alter the accuracy of the research and aids in clearer communication of the content, such a practice should be deemed acceptable. However, in our study, we found that 3 of the 30 ChatGPT-generated abstracts contained incorrect conclusions.

Finally, Wang and Zhou are right in cautioning against complete reliance on AI language models for labor-intensive tasks, which may lead to a lack of critical thinking and the production of low-quality or erroneous articles. However, their perspective differs from the purpose of our study. We aimed to assess the applicability of an AI model in generating abstracts for basic preclinical research. We found that the quality of ChatGPT-generated abstracts for basic preclinical research was suboptimal and that accuracy was not 100%.

## Abbreviations

AI: artificial intelligence
